# Life Satisfaction and Adaptation in Persons with Parkinson’s Disease—A Qualitative Study

**DOI:** 10.3390/ijerph18063308

**Published:** 2021-03-23

**Authors:** Lina Rosengren, Anna Forsberg, Christina Brogårdh, Jan Lexell

**Affiliations:** 1 Department of Health Sciences, Lund University, S-221 00 Lund, Sweden; anna.forsberg@med.lu.se (A.F.); christina.brogardh@med.lu.se (C.B.); jan.lexell@med.lu.se (J.L.); 2 Department of Neurology, Rehabilitation Medicine, Memory Disorders and Geriatrics, Skåne University Hospital, S-221 85 Lund, Sweden; 3 Department of Thoracic Surgery, Skåne University Hospital, S-222 85 Lund, Sweden

**Keywords:** adaptation, psychological, Parkinson’s disease, quality of life, qualitative research, self-management, sense of coherence

## Abstract

Persons with Parkinson’s disease (PD) need to adapt to their progressive disability to achieve and maintain a high degree of life satisfaction (LS), but little is known about the meaning of LS and adaptation. This study aimed to gain an in-depth understanding of the meaning of LS and adaptation in persons with PD. Open-ended in-depth interviews were performed with 13 persons diagnosed with PD, 9 women, 3 men, and one non-binary person (mean age 54 years, mean time since diagnosis 3.4 years). The interviews were analyzed using a phenomenological–hermeneutic approach. The participants were in the process of adapting to their new health situation. There were two quite distinct groups: one that adapted through acceptance and one that struggled to resist the disease and the profound impact it had on their lives. The thematic structural analysis covers eight themes illustrating the meaning of LS and adaptation, through either acceptance or resistance. Adaptation to PD involves a transitional process characterized by either acceptance or resistance, which influences a person’s LS. Acceptance makes LS possible, whereas resistance constitutes a behavioral barrier to adaptation and LS. Rehabilitation professionals need to understand this individual process to be able to support a person with PD to reach and maintain a high level of LS. Understanding the link between LS and adaptation can support rehabilitation professionals to provide targeted interventions for people with PD.

## 1. Introduction

Parkinson’s disease (PD) is one of the most common progressive neurological disorders and affects about 1% of adults over the age of 60 years [1]. PD is complex, characterized by motor and non-motor symptoms, and highly individual in its progression. Regardless of the course, PD can affect a person’s ability to perform and participate in daily activities [2] and ultimately lead to a reduced life satisfaction (LS) [3,4,5].

LS is a global term describing a person’s subjective content with life. It is defined by Diener as “a global assessment of a person’s quality of life according to his own chosen criteria” [6]. LS is an important goal in rehabilitation for persons with life-long neurological disabilities [7] and is dependent on the degree to which expectations are fulfilled and the person’s own adaptation process [8,9]. In fact, successful adaptation is often linked to a high level of LS [9,10,11]. It is therefore important to be able to assess LS and understand how LS is associated with factors related to the disease itself and personal factors, i.e., features of the person’s life that are not part of their health condition. Moreover, as LS is a highly subjective experience, it is important to understand the meaning of LS in persons with PD from their own perspective.

We have initiated a project with the aim to obtain a broad and in-depth understanding of LS in people with mild to moderate PD. In the first study [12], we evaluated the psychometric properties of the Satisfaction with Life Scale (SWLS), one of the most commonly used rating scales for LS. We found it to be a sound and suitable tool to assess LS in people with PD [12]. In the subsequent study, we investigated, for the first time, the association between LS, as assessed with the SWLS, and various factors related to the person’s PD and the resultant disability [4]. We found that sense of coherence (SOC), a salutogenic concept by Antonovsky [13], was the factor most significantly associated with LS. A strong SOC was positively related to a high LS [4]. The strong association between LS and the personal factor SOC shows the importance of understanding, managing, and feeling motivated as part of an adaptation process to the new life situation with PD, thereby reaching a high level of LS.

Since PD is a progressive disorder, one can assume there is a strong need for persons with PD to continuously adapt to their new life situation in order to reach and maintain a high level of LS. It is therefore important to understand their inner perspective with regard to their view on PD, and the meaning of LS and adaptation [14]. To reach such an understanding, the most suitable approach is to apply a qualitative research design [15]. Qualitative research deals with experiences, perceptions, and meaning, thereby allowing an in-depth understanding of a person’s inner perspective [16]. To the best of our knowledge, no qualitative study has been undertaken of LS and adaptation in persons with PD.

The aim of this study was to explore the meaning of LS and adaptation in persons with mild to moderate Parkinson’s disease.

## 2. Materials and Methods

### 2.1. Research Design

This study was carried out with a phenomenological–hermeneutic approach based on the philosophy of Ricoeur [17] to explore the participants’ lived experience. This empiric holistic approach is chosen to explore the meaning underneath the surface and thereby gain an in-depth understanding of the meaning of LS and adaptation in persons with PD. The analysis was performed using a phenomenological–hermeneutic method developed by Lindseth and Norberg [18].

### 2.2. Setting and Participants

Recruitment of participants was initially performed through the regional PD patient organization in the south of Sweden. To obtain a nationwide distribution, we subsequently approached the national patient organization for persons with PD. The inclusion criteria were a verified diagnosis of PD (ICD10:G20) for at least one year, age below 65 years, and being able to participate in the interview; we did not exclude persons with mild cognitive impairment. The sampling was purposive as we specifically approached persons younger than 65 years. The diagnosis of PD is often made in people under the age of 65 years, and those who are working have a different perspective on life than retired people. Recruiting participants from a patient organization also enabled rich narratives. We aimed to achieve an equal gender distribution; however, it was somewhat difficult to recruit male participants. Finally, the 13 participants comprised 9 women, 3 men, and one non-binary person, with a mean age of 54 years and a mean time since diagnosis of 3.4 years (Table 1). All participants were community-dwelling, ambulating, independent in Activities of Daily Living (ADL), and most of them (n = 10) were still working. A majority of the participants were in Hoehn and Yahr stage 1 or 2, and one participant in stage 3, and were thus considered to have a mild to moderate PD. None of the participants were judged to be clinically depressed or having any neuropsychological problems, such as memory difficulties, attention and concentration problems, or fatigue that prevented them to fully participate in the interviews.

### 2.3. Data Collection

Before the interviews, the participants received a letter with information about the study, an informed consent form, and a questionnaire including socio-demographics. All interviews were performed by the first author during a 6-month period (October 2019 to March 2020) at a place chosen by the participants, e.g., their own home (n = 8) or by digital techniques (n = 5).

The interviews consisted of reflective, open-ended questions, and were digitally recorded and transcribed verbatim soon afterwards. All interviews were conducted in Swedish. The questions, posed in Swedish and here translated to English, explored the lived experiences of suffering from PD and perspectives on LS, such as “Can you please tell me what PD means to you?” and “Can you please tell me about when you feel satisfied with life?”, with follow-up questions like “Can you please describe…?” or “Can you please tell me something more about…?”. During the interviews, the information received was recapitulated to give the participants a chance to clarify or develop further. The interviews lasted on average 67 min (range 42–110 min) and resulted in 222 pages of transcribed text. After 13 interviews, we were convinced that we understood what we saw, could identify the relevant forms of the results, and that it appeared culturally consistent. Thus, we decided that we had ensured saturation and therefore terminated the data collection.

### 2.4. Data Analysis

In the phenomenological–hermeneutic method developed by Lindseth and Norberg [18], the interpreter moves from a naïve reading to an in-depth understanding [19]. In the first step, the naïve reading, the researchers read the interviews several times in order to grasp the meaning. In the second step, the structural analysis, the text was divided into meaning units and then condensed, brought together, and sorted into subthemes and themes to capture the meaning of adapting to PD and LS. In the third and final step, the comprehensive understanding, the researchers once more read the interview texts as a whole, and reflected on it together with the naïve reading. Themes were validated in relation to the research question and the context of the study [18]. The interpretation of the findings reflected the researchers’ pre-understanding as members of a rehabilitation team, i.e., two physicians, a nurse, and a physiotherapist working at a rehabilitation clinic or with persons with life-long disabilities or chronic conditions in both in-patient and out-patient specialist care.

## 3. Results

### 3.1. Naïve Understanding

The naïve understanding revealed that the participants were in the process of adapting to PD with or without pronounced symptoms and various consequences in their everyday life. There were overall two quite distinct groups: one with participants that proceeded with their adaptation by general acceptance, and one that mostly struggled to resist the disease itself and its profound impact on their lives. Therefore, the thematic structural analysis (Table 2) comprises eight themes illustrating the meaning of LS and adaptation as two sides of the same coin, either through accepting

*“You have to learn to live with your disease and if it demands a certain kind of food or that you have to do things differently, then that is what you have to do to live your life. So, I guess that if Mr Parkinson has decided that I must work out every day, then that’s what I do. He can decide that and I decide the rest.”*

(I5)

or resisting

*“It’s so much, I mean you get so tired of this. One day it’s this and the other day it’s that. You’re in pain or you turn into a victim… or no… it’s just a lot.” *

(I3)

Adaptation started when awaiting the definite diagnosis and went on continuously, moving back and forth in a constant process of change.

*“But of course, it’s still a process because I’m not done yet, I’m far from done. Maybe I never will be when living with a disease, that can always keep changing.”*

(I11)

### 3.2. Structural Analysis

#### 3.2.1. Awaiting the Diagnosis

Being in acceptance meant realizing that something is wrong with your body and at the same time not knowing the reason for the physical changes. Being in this pre-diagnostic phase meant facing the reality by preparing for the worst and at the same time trying to continue with an everyday life as normal as possible.

*“I was in such a bad shape, so I thought I’m not going to survive this, and I have two kids. I had this feeling, shit it’s over.”*

(I5)

For those in resistance the uncertainty was a torture, making it impossible to relax. They were unable to function in everyday life, felt paralyzed, and put life on hold.

*“You’re counting the days… you’re putting life totally on hold and simply waiting. I mean, there is nothing more important than seeing a neurologist. At least, that’s how it was for me and I don’t know if I’m over sensitive or something. But that’s how it was for me.”*

(I11)

#### 3.2.2. Facing the Diagnosis

Receiving the final diagnosis meant relief and confirmation of previous fears. They had suspected that the diagnosis would be PD or something similar. It was a relief to receive an explanation of the various symptoms, that they had not imagined the whole thing, and to realize that the immediate threat to life was exaggerated. After accepting that life would continue, new questions emerged about future consequences and new lifestyle demands.

*“It was like, I don’t have a brain tumor, I’m not going to die! And then at the same time, I have Parkinson, I’m going to live with that for the rest of my life. It was a lot of mixed emotions, but the first feeling was, I’m not going to die, I’m going to live, I have my whole life in front of me and then, wait a second… what does that mean? What’s going to happen to me while living with Parkinson? So that was a new thing to confront.” *

(I5)

For the participants in resistance the diagnosis meant a shock, and for those with a clear pre-understanding of the meaning of PD it was harder to face the reality, expecting the worse. They were painfully aware of the progression of their disease without knowing how to cope. Knowledge or experience of the disease could make it harder to face the diagnosis.

*“It was the beginning of the end of life. Through my work in home care I’ve seen what happens at the end of life and it’s very sad. Home care means that you’re limited and it’s hard and depressing. Maybe I’m not there now but it’s coming. I know what awaits me.” *

(I13)

#### 3.2.3. Feeling Ashamed

Having a chronic, and possibly, disabling disease meant feeling ashamed of one’s symptoms, e.g., trembling hands or inability to master stressful situations. Facing the diagnosis as part of adaptation was not only a matter of one’s own fears and expectations. It also meant addressing expectations from others. Sharing the illness experience was a way of slowly accepting and adapting to the disease, leading to a sense of serenity.

*“Well, I can say that I accepted it in a logical sense when he [the neurologist] told me, because I realized that it was the truth, I mean it sort of fitted the way I felt. His information became a sort of model explanation of the reality I was in… but then it takes a long time to accept the whole thing emotionally. To accept this situation and have the courage to stand up for myself. I’m not Parkinson but Parkinson is a part of me and it’s nothing to be ashamed of. I don’t have to hide it from others. It was important to reach that point”.*

(I9)

When resisting, being ashamed meant dealing with the stress of hiding symptoms from others, e.g., trying to sit on one’s hand when it was shaking. It was difficult to share information about the disease due to fear of being rejected by friends and colleagues or by looking different compared to before. When attending a job interview there was a fear of not being considered if the disease was mentioned. Instead, they hid the facts while dealing with enormous stress inside. The shame led to isolation and avoidance of social situations as the disease affected self-esteem and created a sense of reduced self-worth.

*“And I’m afraid that I’ll be shaking when taking a walk outside. I don’t want to look different while I’m outside. I really don’t want to depend on a walker when I’m 65.”*

(I13)

#### 3.2.4. Approaching the New Life Situation

When in acceptance, the participants realized that a changed everyday life was needed and faced their own responsibility. They also believed that they had the ability and motivation to make the required adjustments. It included taking control over the situation, being in charge of one’s life, and not handing it over to healthcare professionals. Furthermore, they understood that the necessary adjustments regarding physical exercise and food were not a quick fix, but time consuming and demanding with no shortcuts. Accepting the disease meant facing reality and being responsible.

*“I decided to make it work by assuming my responsibility. I must be in charge of this process because nobody else can. As soon as you hand it over to someone else, a physician for example, you fail. You must take control. That’s the only option, unfortunately. It’s a difficult process, but you have to do it.”*

(I11)

Instead of facing reality, the participants who resisted thereby avoided approaching their new life situation and the required adjustments. They felt helpless and viewed themselves as a victim of the disease. Feeling incapable or paralyzed, they hoped for external assistance from spouses or significant others. They focused on problems, losses, and limitations and found it impossible to accept that they had a responsibility to adjust.

*“I actually hide my head in the sand. The thing is I only know what I have seen at work. I can’t even read about Parkinson’s disease. I have problems with my thyroid as well and I know all about that, I mean absolutely everything. But I know nothing at all about Parkinson. I simply don’t have the energy to read about it.”*

(I13)

#### 3.2.5. Being in Transition

Adaptation to PD meant being in transition—from a person without a chronic disease via something unknown, to being on the path to a new normality and balance in life. Some of the participants acknowledged the restraints and balanced their expectations while focusing on problem solving. A key challenge was accepting the fact of being far more sensitive to stress and developing new strategies to master stressful situations. They realized that a day will come when it will no longer be possible to ride a bike due to balance problems, but the main goal was not allowing the disease to dictate the rules in everyday life.

*“I’m not going to climb Kilimanjaro or Machu Picchu. So, a lot of things I dream of doing might need re-evaluation as they involve too much stress … You simply must slow down. Going on the Hurtigruten [long boat trip] and things like that.”*

(I1)

While in transition, they focused on positive things and achieved balance by adjusting to the limitations, accepting setbacks, and engaging in meaning-making. In addition, they focused on being kind to themselves, maintaining hope, and reformulating previous goals in order to avoid disappointments.

*“You can never lose hope, you know. That’s when you can’t cope. I have switched focus from being healthy to try to be as healthy as possible with Parkinson’s disease. So, it has limited my future a bit, it’s more framed by the disease. I guess I like to know when I’m making progress. When I succeed in stopping the decline. That’s the only thing. It’s my biggest goal actually, not getting worse.”*

(I11)

Knowledge of the progress of the disease made them use time wisely and prioritize successfully. Support from their employer meant that they could adjust their work situation. Establishing a balance enabled them to cope with the stress, which had a major impact on all the participants. Positive feedback on one’s efforts and seeing progress were important for maintaining motivation. Stubbornness was a useful characteristic for facilitating ambitious self-care.

*“And actually, my arm started to oscillate a little after six weeks of training and then I became so happy. It was the happiest moment. Then I realized that I can do something myself. And now, my arm is oscillating normally again.”*

(I11)

For those in resistance, the transition was delayed for several months due to the shock of the disease.

*“So, it is not even worth living, it was such a shock. Just that it felt so… that you couldn’t influence it yourself at all, with the help of a healthy lifestyle or something, that it was just going to get worse. It was devastating, the greatest shock I’ve had in my whole life. I was totally under the weather for maybe two months.”*

(I11)

There was a strong focus on lack of control, losses, and being disappointed, while painfully aware of one’s limitations. The whole situation was resisted in an energy consuming process, where they struggled to find balance, wasted time, and felt incapacitated. The participants in this group experienced profound difficulties in mastering stress and used their energy on things that drained them instead of giving strength. Sitting at home, too tired to act or expecting the day to be bad led to isolation.

*“If it’s a good day then it’s all right and if it’s a bad day it’s not even worth going there at all, it kind of goes up and down and it’s hard to understand it… It’s really hard to plan anything.”*

(I8)

Stubbornness was an asset for the group that had accepted but became a barrier for those who resisted by maintaining a negative approach to life leading to missed opportunities. Instead of accepting the ups and downs as a natural part of life, the dynamics of the disease became difficult to understand.

*“It sounded great you know, rehab, because I have pain. I was accepted for rehab, but I work 75 percent in home care, I mean I just didn’t have the energy… I was supposed to go twice a week and it was so hard, you know I have to walk the dog too, I mean I got completely stressed out, so I had to quit.”*

(I13)

#### 3.2.6. Adjusting to the Medication

Being diagnosed with PD always means starting new medication, as dopamine replacement therapy is part of the disease. The medication was a new and complex factor to cope with. Scheduling and evaluating the doses and effects by trial and error was a way of understanding how the medication worked. They could then use this strategy as a tool to feel well in everyday life.

*“I have to take my medication at the right time, I have to make sure to eat right, I have to work out. But other than that, I live as before, I mean… I actually feel even better now because I eat right.”*

(I5)

For those in the resistance group, the strict scheduling necessary to obtain the best effect of the medication was considered limiting and restrictive.

*“It’s really difficult to plan anything. And then I have to remember to take my pills every third hour throughout the day, so that’s something you have to remember all the time and it’s, yes it’s really difficult to get into a good rhythm.”*

(I8)

The downside of the medication was the potential side-effects, which were sometimes more severe than the symptoms of the disease itself. The response fluctuations made them insecure and led them to avoiding meaningful activities. The side-effects made them distrust the medication and caused a sense of insecurity.

*“When the dopamine wears off, I feel like a turtle.”*

(I9)

#### 3.2.7. Playing the Patient Role

Besides dealing with their own expectations and the perceived expectations of others, adapting to PD also meant entering the healthcare system in the role of a patient. For those in the acceptance group, healthcare was viewed as a compliment to self-care. Continuity of care was the key to a positive experience of being a patient.

*“Six weeks after the first appointment at the health care center I got the hospital appointment to see my neurologist who is still there for me. I have the best healthcare in the world.”*

(I10)

On the other hand, the experience could be extremely negative when resisting everything that concerned the disease and its treatment. Instead of being supportive, the experience of healthcare could be a lack of availability, which made the patients insecure. They had to phone multiple times to obtain new prescriptions for their medication. If they did not get it in time, they borrowed medication from friends with PD as an extra self-care strategy.

*“… and then it’s the healthcare system you know. It kills you. You have to be healthy to be ill, because you have to struggle for everything.”*

(I3)

Lack of continuity of care including serious difficulties to get in contact with the neurology clinic made the patients disappointed and resigned. Not being taken seriously evoked a sense of being neglected and viewed as an object or a diagnosis instead of a capable human being.

*“The starting point was always his [the neurologist’s] perspective. I really wished for someone who adopted my viewpoint, paid attention to how it works for me and asked about my family, what causes stress in my life. I’m a human being you know. But for him I was a diagnosis and that’s not the same thing.”*

(I9)

Mistrust of healthcare could either lead to extensive self-care or a feeling of insecurity. The distrustful caring relationships could be caused by the patients feeling questioned, experiencing prejudices, or meeting staff who lacked knowledge about the disease.

*“The same year I was diagnosed with Parkinson’s, I went to a neurologist who told me that my shakiness was psychosomatic… this thing of always being questioned because I have the wrong disease for my age and then I have other chronic conditions. I often hear that it’s not possible. I can’t have both.”*

(I12)

#### 3.2.8. Being the Same but Different

Having PD changed the perspective on life. One’s position and role were altered and life expectancies transformed, causing both grief and the courage to make changes.

*“It’s a kind of grief, in some way you’re mourning for the life you thought you had, and you feel like, you won’t be able to do this and that, and then suddenly you realize that yes I can, there’s nothing stopping me.”*

(I5)

When in acceptance you cling to your previous identity but accept that you might have a new role in life. Knowledge of the progression of the disease could make it easier to focus on the present and try to make the best of it. Instead of procrastinating they handled situations more efficiently. Not being defined by the disease was crucial.

*“First of all, I am not Parkinson’s Disease, I’m a person with an active life… I’m a mother, a friend, a LARPer [live action role player], a literature scholar… Indeed, I have Parkinson 24/7 as well, but that’s not what defines me!”*

(I12)

The illness and inherent uncertainty about the future facilitated a positive approach to life and it was easier to remain constructive and enjoy life as it is. It also meant accepting financial changes. Focusing on the present and accepting the situation evoked a sense of serenity.

*“Life is more... live as you are today because you never know what tomorrow will bring. And I wish more people would do that. You don’t have to become ill to realize that you have to enjoy life.”*

(I5)

On the other hand, some persons felt lost and mourned the loss of their role. The change was especially painful for those with a strong work identity.

*“It’s a misery, I have such a strong occupational identity. Most of my friends don’t call me N.N, they call me by my occupation. That says a lot… so it’s kind of a big fall.”*

(I9)

The awareness of the progression of the disease and their uncertainty made them focus on the future instead of living in the present. It could lead to dwelling on the problems and making comparisons when feeling incapable of managing their life situation. They constantly worried about what will happen to their children and family members. In addition, the financial aspect of living with a chronic disease led to a great deal of stress and could feel like a disaster.

*“How am I supposed to fit everything into my everyday life? And I also must find myself. And I don’t manage to apply for a job. And if I am unable to work? I must be on sick leave to find myself, to settle down. Maybe in two years I can work full time again, I don’t know, but today I can’t.”*

(I10)

### 3.3. Comprehensive Understanding

Our comprehensive understanding of the findings is that living with PD is a transitional adaptation process facilitated by either an individual ability to accept the disease and its impact on everyday life or be delayed and obstructed by a personal resistance towards the disease and its consequences. Accepting the circumstances made it easier to adapt in all areas of life, to interact with healthcare professionals, and to find useful coping strategies in relation to side-effects of the medication and the necessary scheduling of daily activities. In contrast, resisting the disease caused a profound barrier where disappointments, bitterness, and uncertainty about the future disabled them even more than the symptoms. The relationships with healthcare professionals were characterized by absence, mistrust, and lack of continuity. Resisting is energy consuming and exhausting, whereas accepting preserves energy for various self-care activities and eventually leads to moments of serenity and contentment.

When interpreting these findings in the light of LS, it seems obvious that adaptation through acceptance eventually leads to LS, whereas resistance hinders this state. Those with a profound ability to accept described an ongoing adaptation and eventually they experienced higher LS, as defined in terms of the degree to which a person positively evaluates the overall quality of their life [20]. Overall, their feelings about life were positive and this evaluation was global rather than grounded in any specific point in time or domain [21].

## 4. Discussion

This is, to the best of our knowledge, the first study to explore the meaning of LS and adaptation in persons with mild to moderate PD. In this qualitative study with a phenomenological–hermeneutic approach [18], the core findings are that LS is reachable through adaptation to the new life situation with PD and that adaptation involves a transitional process based on either acceptance or resistance. Acceptance makes it possible to achieve LS, while resistance constitutes a behavioral barrier to both adaptation and LS. The individual participants could, of course, be in resistance in some situations when living with PD and in acceptance in other situations. However, in this qualitative study, we have explored and dichotomized the meaning of their lived experiences, but not the participants themselves.

Our findings support previous suggestions that adaptation is the key to a higher LS [9,10,11]. However, none of the previous studies explain how this connection is constructed. In theory, one can imagine that adaptation can both improve one’s achievements and adjust one’s expectations, which in turn improve LS as a subjective evaluation of one’s life situation. Diener et al., who conceptualized LS [22], raised the question of what makes some persons adapt while the adaptation process does not work for others. Understanding the link between LS and adaptation and the process that underlies adaptation is therefore an important area for future research.

A noticeable finding was the obvious difference in the person’s adaptation process—being either in acceptance, which facilitates the adaptation or being in resistance, which inhibits the process (cf. Table 2). A review of qualitative studies described how individuals adjusted to having PD, the difficulty coming to terms with the disease, and primarily not accepting what had happened [23]. Accepting the diagnosis enabled people to accept the different meaning it had for their life, including social and vocational identities. The role of acceptance for adaptation has been studied in palliative care [24], where it was described how acceptance could appear more challenging for some persons. The authors described it as a shift where some individuals adjusted the relationship between capability and utility to allow them to achieve greater utility from a poorer capability state [24]. This explanation is similar to the results of the present study, where the persons in acceptance focused on abilities and possibilities rather than limitations. As no study has specifically explored the link between LS and adaptation, our study contributes with new knowledge regarding the meaning of LS and adaptation in people with PD.

Our results can be interpreted and understood from several perspectives. Based on our previous studies of LS in people with PD [4,12], it is appealing to view the results in the present study from a salutogenic perspective [13]. Participants that were in acceptance appeared to cope with their new life situation more efficiently than the participants in resistance. Participants in acceptance seemed to have the ability to understand, handle, and were motivated when dealing with stressful events and problems arising as a result of their PD, the core concepts of a strong SOC. According to Antonovsky, SOC develops during childhood and adolescence, and is strengthened by resources such as wealth, intelligence, social support, and cultural stability [13]. SOC indicates how well a person handles stress that is a result of a disorder or a disability [25]. A person with strong SOC views stressful events as less threatening compared to people with a weak SOC [26]. The strong association between SOC and LS, described in our previous study [4], shows the importance of understanding, managing, and feeling motivated as part of an adaptation process to a new life situation with PD. Thus, the level of SOC is hypothesized to be one personal factor that explains why some persons with PD adapt to their new life situation, whereas the adaptation process is less successful for others. To the best of our knowledge, the association between LS, adaptation, and SOC has not been studied in PD.

### 4.1. Clinical Implications and Future Research

To reach and maintain a high degree of LS, healthcare professionals must focus on how persons with PD react when they receive the diagnosis. They should then follow the adaptation trajectory of persons with PD and as soon as possible identify those in resistance who adopt a reactive pattern of behavior as it will delay the necessary adaptation and constitute a persistent barrier to LS. This could also improve outcomes of various interventions. One study, exploring the lived experience of deep brain stimulation (DBS), showed that the more persons with PD felt alienated by their illness, the more they experienced post-operative self-estrangement [27]. The authors also stated that the notion of self-estrangement seems to exist in association with loss of control and distorted perception of capacities, qualitative characteristics that were common also among our participants being in resistance.

To understand how the person is comprehending the situation, it is paramount that we ask a simple, yet important, question, “How do you understand this particular situation?”, as a hermeneutic approach to persons with PD. This could be a starting point to recognize a person with PD in acceptance or resistance (as described in Table 2) and then support them through the adaptation process.

From a rehabilitation research perspective, this study emphasizes the need to understand the process that underlies adaptation in order to support the persons with PD to go through this process. To be able to support their adaptation towards a high level of LS, we need to understand how this process is carried through, in other words—how they do it and why? Further studies should therefore focus on understanding the process of adaptation for reaching and maintaining a high level of LS. This will enable rehabilitation professionals to develop interventions for persons with PD that enhances LS over time.

### 4.2. Methodological Considerations

It was difficult to recruit men for the interviews; thus, a limitation is the somewhat uneven gender distribution. However, one participant self-identified as non-binary, which we considered a strength of the purposeful sampling. Only recruiting through patient organizations could be viewed as a limitation. On the contrary, it was performed to include participants who would provide rich narratives. The authors pre-understanding, as members of a rehabilitation team and working with persons with life-long disabilities, is also a strength for the interpretation of the participants’ lived experiences. Since all the participants were considered to have a mild to moderate PD, were below the age of 65 years, and were Swedish, transferability is limited to other severities of PD, other age groups, and other ethnical and cultural contexts. All quotations were translated from Swedish to English and validated by a native speaking professional English translator to fully capture the original meaning and intention by the participants.

## 5. Conclusions

Adaptation to Parkinson’s disease involves a transitional process characterized by either acceptance or resistance, which influences a person’s LS. Acceptance makes LS possible, whereas resistance constitutes a behavioral barrier to adaptation and LS. Rehabilitation professionals need to understand this individual process to be able to support a person with PD to reach and maintain a high level of LS. Understanding the link between LS and adaptation and the process that underlies adaptation can support rehabilitation professionals to provide targeted interventions for people with PD.

## Figures and Tables

**Table 1:** Characteristics of the 13 participants with Parkinson’s disease.

Gender	
Men	3
Women	9
Non-binary	1
Age (years)	
Mean, SD (range)	54, 4.9 (47–62)
Years with symptoms	
Mean, SD (range)	7.3, 5.7 (3–24)
Years since diagnosis	
Mean, SD (range)	3.4, 1.7 (1–7)
Marital status	
Single, n	3
Married/cohabitating, n	10
Vocational status	
Working ^1^, n	10
Old age pension, n	2
Disability pension, n	0
Other ^2^, n	1

^1^ Includes working full time, part time sick leave, and looking for work. ^2^ Has chosen to not work or look for work.

**Table 2:** Structural analysis of the meaning of adapting to Parkinson’s disease.

Main Themes	Sub-Themes	
	Acceptance	Resistance
Awaiting the diagnosis	*Preparing for the worst* *Continuing with everyday life*	*Uncertainty* *Putting life on hold*
Facing the diagnosis	*Feeling relieved*	*Being painfully aware of the progression of the disease*
Feeling ashamed	*Sharing the illness experience*	*Isolating and avoiding social situations* *Feeling less worthy* *Avoiding*
Approaching the new life situation	*Accepting that change is necessary* *Accepting one’s own responsibility* *Believing in one’s ability*	*Feeling like a victim of PD* *Hoping for external assistance* *Feeling impotent*
Being in transition	*Acknowledging the restraints* *Balancing expectations* *Establishing balance*	*Experiencing losses* *Being painfully aware of the limitation* *Struggling*
Adjusting to the medication	*Scheduling and evaluating the doses and effects* *Understanding the treatment*	*Feeling restrained and limited* *Doubting the treatment*
Playing the patient role	*Performing extensive self-care* *Experiencing continuity*	*Feeling disappointed* *Mistrusting the professionals* *Lacking continuity*
Being the same but different	*Having the courage to change* *Keeping one’s identity* *Finding a new role in the family* *Living in the present* *Experiencing serenity* *Accepting the changes in work and finances*	*Feeling lost* *Mourning the loss of one’s role* *Dwelling on problems and comparing* *Worrying about the work and financial situation*

## Data Availability

All data were archived according to the Swedish Act concerning the Ethical Review of Research Involving Humans to attain confidentiality and are available upon reasonable request.
